# Cardiorespiratory Fitness Decreases High-Sensitivity C-Reactive Protein and Improves Parameters of Metabolic Syndrome

**DOI:** 10.7759/cureus.63317

**Published:** 2024-06-27

**Authors:** Hildemar Dos Santos, Micheline A Vargas, Josileide Gaio, Princess-Lisa Cofie, Wenes P Reis, Warren Peters, Lee Berk

**Affiliations:** 1 Preventive Care, Loma Linda University School of Public Health, Loma Linda, USA; 2 Obesity Research, Loma Linda University School of Public Health, Loma Linda, USA; 3 Research Affairs, School of Allied Health, Loma Linda University Medical Center, Loma Linda, USA

**Keywords:** bruce protocol treadmill test, metabolic syndrome, c-reactive protein, inflammation, atherosclerosis, : exercise

## Abstract

Aim: To evaluate the relationship between cardiorespiratory fitness (CRF), expressed as maximal oxygen uptake (ml.kg^-1^.min^-1^), metabolic syndrome (MetS), and high-sensitivity C-reactive protein (hs-CRP), a marker of systemic inflammation.

Methods: The relationship between CRF, MetS, and hs-CRP was examined in a cohort of 173 men and women. CRF was evaluated using a Bruce protocol treadmill test and measured as estimated maximal oxygen uptake (VO_2 _max). Participants' physical activity status was self-reported. Plasma hs-CRP levels were measured using a standardized immunoassay, and the diagnostic criteria for MetS were based on guidelines established by the International Diabetes Federation (IDF).

Results: An inverse association was observed between hs-CRP levels and estimated VO_2_ max (p<0.01). Additionally, hs-CRP increased linearly with the number of MetS criteria present (p<0.01), while the estimated VO_2_ max decreased as the number of MetS criteria increased (p<0.01). Moreover, higher estimated VO_2_ max correlated with increased self-reported physical activity levels (p<0.01). Notably, participants engaging in two to three hours of exercise per week had hs-CRP levels ≤2.5 mg/L (p=0.018), considered a low-to-moderate risk range.

Conclusion: Higher CRF, reflected by an estimated VO_2_ max, ≥45 ml/kg/min, is associated with lower hs-CRP levels and fewer MetS criteria. Additionally, regular physical activity, corresponding to higher VO_2_ max, appears to reduce systemic inflammation and ameliorate MetS risk factors. These findings support the mechanisms by which improved CRF and exercise may lower the risk of cardiovascular diseases (CVD) and type 2 diabetes (T2DM).

## Introduction

The leading cause of death in the United States (US) is cardiovascular disease (CVD) [1]. Today, it is well known that insulin resistance plays an integral role in CVD etiology [2,3]. Certain CVD risk factors appear to be exacerbated by insulin resistance. According to the International Diabetes Federation (IDF) guidelines, metabolic syndrome (MetS) is marked by the presence of central obesity (if BMI > 30 kg/m^2^, then central obesity can be assumed) plus any two of the following risk factors including elevated serum triglycerides (TG) level (≥ 1.7 mmol/l (150 mg/dl)), low high-density lipoprotein (HDL) cholesterol (< 1.03 mmol/l (40 mg/dl) in males and < 1.29 mmol/l (50 mg/dl) in females), elevated blood pressure (systolic BP ≥ 130, diastolic BP ≥ 85 mmHg), and impaired fasting plasma glucose (FPG) (≥ 5.6 mmol/l (100 mg/dl)) [4].

MetS is also strongly linked to an elevated likelihood of developing conditions such as CVD, diabetes mellitus (DM), and various types of cancers [5]. According to the US National Health and Nutrition Examination Survey (NHANES) conducted between 2011 and 2018, there was a notable rise in the prevalence of MetS in the US in adults aged ≥20 years [6]. The rates were reported as 37.6% during 2011/2012 and increased to 41.8% in 2017/2018 [6]. The risk of CVD appears to be increased by two-fold and type 2 diabetes (T2DM) by five-fold [7]. Hence, the IDF report emphasizes the significance of preventive therapies for these individuals.

Engaging in regular physical activity has been shown to have notable impacts on the immune system and may offer protective benefits against CVD and DM [8]. Physical activity is also considered an important determinant of MetS [9]. All levels of MetS, DM, and CVD are thought to involve inflammation. Physical activity may reduce risk, at least in part, by modifying the inflammatory process. Recent studies have demonstrated an inverse relationship between inflammatory markers, such as high-sensitivity C-reactive protein (hs-CRP), and physical activity [10,11]. 

Elevated hs-CRP appears to be an independent predictor of both CVD and DM. Recent evidence also suggests that hs-CRP is positively associated with all MetS characteristics [12,13]. Cardiorespiratory fitness (CRF), an indicator of the body's ability to supply oxygen to the muscles during sustained physical activity, has been shown to have protective effects against CVD and MetS [13]. While the benefits of physical activity on metabolic health and inflammation are well-established, there is a need for a comprehensive investigation of the interplay between CRF, MetS, and hs-CRP levels. Existing studies have primarily focused on individual aspects of this relationship, leaving a gap in our understanding of the overall dynamics.

In this study, we aimed to evaluate the relationship between CRF, expressed as maximal oxygen uptake, VO2 max (ml.kg^-1^.min^-1^), MetS, and hs-CRP levels in a unique population of individuals who underwent preventive medicine health exams. By utilizing a comprehensive set of clinical measurements, including a Bruce protocol treadmill stress test, blood analysis, and detailed personal wellness profile questionnaires, we sought to provide insights into the mechanisms by which CRF and exercise may reduce the risk of CVD and DM through the modulation of inflammation and MetS characteristics.

## Materials and methods

This study was conducted at the Loma Linda University, Loma Linda, California, United States. The study was approved by the Loma Linda University Institutional Review Board (IRB # 55168) and was in accordance with the ethical standards of the institutional and/or national research committee and the 1964 Helsinki Declaration and its later amendments or comparable ethical standards [14]. All participants gave their informed consent prior to their inclusion in the study.

Study sample

Study participants were selected from a pool of 1,072 men and women from the Center of Health Promotion (CHP) at Loma Linda University. Participants were included in this study if they: (i) underwent a preventive medicine health exam at the CHP, which included blood pressure screening and blood analysis (hs-CRP, lipid profile, and fasting blood glucose), (ii) completed the Comprehensive Personal Wellness Profile (PWP) questionnaire regarding their medical history and lifestyle health practices (Wellsource Inc., Tigard, Oregon, United States), (iii) reached ≥ 85% of age-predicted maximal heart rate on a Bruce protocol treadmill stress test, and (iv) were able to give informed consent. Excluded from the study were participants who did not reach at least 85% of their age-predicted maximal heart rate during the Bruce protocol treadmill stress test, participants who were unable to provide informed consent, participants with certain medical conditions and/or disabilities that may have prevented them from safely completing the treadmill stress test or other required assessments, participants taking medications that could potentially interfere with or influence the study measurements such as anti-inflammatory drugs or medications for metabolic disorders, participants with known CVDs, T2DM, or other chronic conditions that could affect their metabolic profiles or inflammatory states, as the study aimed to examine a relatively healthy population, and participants who were pregnant or breastfeeding. A total of 173 subjects met the inclusion criteria and were included in the study.

Clinical measurements

CRF level was assessed via VO2 max estimated from a Bruce protocol treadmill stress test. The PWP questionnaire was used to collect information regarding the participant’s medical history and lifestyle behaviors such as smoking habits, alcohol intake patterns, eating habits, stress and coping patterns, social health, and physical activity status. Several questions were asked regarding the participant’s physical activity status. 

Participants were asked about their current physical activity status under the headings of (i) no regular exercise program, (ii) occasionally walking for pleasure, (iii) regular exercise in work or recreation requiring modest physical activity such as golf, yard work, calisthenics, up to one hour per week, (iv) regular exercise in work or recreation requiring modest physical activity such as golf, yard work, calisthenics, more than one hour per week, or (v) more active physical exercise (brisk walking, jogging, swimming). If the participant answered yes to the last question, they indicated how much time was spent engaging in that activity each week. Quest Diagnostics Incorporated (Secaucus, New Jersey, United States) performed blood chemistry analyses. Plasma hs-CRP concentrations were measured with the Dade Bering BN II high-sensitivity immunoassay (Dade Behring, Inc. Deerfield, Illinois, United States). Fasting glucose was performed on the Olympus AU5400 Analyzer (Olympus Corporation, Shinjuku City, Tokyo, Japan). Total cholesterol and triglyceride analyses were performed on the Olympus AU5400 Analyzer, while HDL-cholesterol was performed on a Roche Analyzer (F. Hoffmann-La Roche AG, Basel, Switzerland). Participants had their resting blood pressure measured by auscultation. Standard procedures outlined by the American Medical Association were followed [15]. Height and weight were measured using a standardized physician's balance beam scale and stadiometer. Participants were instructed to remove their shoes and any heavy clothing before measurements were taken. Height was measured to the nearest 0.1 cm, and weight was measured to the nearest 0.1 kg. BMI was calculated as weight (kg) divided by height (m) squared. To ensure reliability, the scale and stadiometer were calibrated regularly, and measurements were taken by trained staff following a standardized protocol.

Definitions

Although the definition of MetS has not been agreed upon internationally, a working definition continues to be used. Our definition utilizes criteria set forth by the IDF (Table 1) [4]. According to IDF guidelines, participants had to meet at least three of the following variables in order to be labeled as having MetS: (i) systolic BP ≥ 130 or diastolic BP ≥ 85 mmHg, (ii) HDL cholesterol <40 mg/dl in males and <50 mg/dl in females, (iii) impaired FPG ≥ 100 mg/dl, (iv) elevated serum TG level ≥ 150 mg/dl, and (5) BMI ≥ 30kg/m^2^.

**Table 1:** International Diabetes Federation (IDF) metabolic syndrome worldwide definition ^*^ Derived from Alberti et al., 2005 [4]. ^†^ If BMI > 30 kg/m^2^, central obesity can be assumed and waist circumference does not need to be measured. HDL: high-density lipoprotein

Risk Factor	IDF Criteria
Central obesity	Waist circumference^*†^: ethnicity-specific plus any two of the following
Raised triglycerides	≥ 1.7 mmol/l (150 mg/dl) or specific treatment for this lipid abnormality
Reduced HDL cholesterol	< 1.03 mmol/l (40 mg/dl) in males < 1.29 mmol/l (50 mg/dl) in females or specific treatment for this lipid abnormality
Raised blood pressure	Systolic: ≥ 130 mmHg or Diastolic: ≥ 85 mmHg or treatment of previously diagnosed hypertension
Raised fasting plasma glucose	Fasting plasma glucose ≥ 5.6 mmol/l (100 mg/dl) or previously diagnosed Type 2 diabetes If > 5.6 mmol/l or 100 mg/dl, oral glucose tolerance test is strongly recommended but is not necessary to define the presence of the syndrome

Due to this study's retrospective design and the lack of waist circumference measurements, we chose to use BMI (≥ 30kg/m^2^) rather than waist circumference as our measure of obesity. The IDF utilizes ≥ 30kg/m^2^ as an assumption of central obesity; thus, waist circumference does not need to be measured.

Statistical methods 

Any missing data variables were imputed using the maximum likelihood method (EM) in SYSTAT version 10 (Systat Software, Inc., Chicago, Illinois). Because the percent body fat variable required over 100 imputations to fill in all of the missing data, it was not used in any analyses. The distribution of hs-CRP, the dependent variable of greatest interest, was positively skewed. Therefore, a log transformation was applied to hs-CRP values for all analyses. Simple regression/correlation analysis was used to evaluate the relationships between log (CRP) and each of the relevant variables: Estimated VO2 max (ml.kg^-1^.min^-1^), metabolic syndrome, and physical activity status.

In the regression analyses, we also controlled for potential confounding variables, including age, gender, smoking status, and alcohol consumption. These variables were selected based on their known associations with CRF, MetS, and inflammation. By adjusting for these factors, we aimed to isolate the independent relationships between CRF, hs-CRP, and MetS severity. The following relationships were explored: (i) hs-CRP and estimated VO2 max, (ii) hs-CRP and MetS characteristics, (iii) Estimated VO2 max and MetS characteristics, and (iv) Estimated VO2 max and physical activity status. Pearson Chi-square analysis was used to determine if a threshold level of physical activity was associated with hs-CRP changes.

## Results

The mean age of the 173 subjects evaluated in the study was 49.5±9.8 years. The clinical characteristics of the study participants are shown in Table 2.

**Table 2:** Characteristics of study participants (N=173) HDL: high-density lipoprotein; LDL: low-density lipoprotein; CRP: C-reactive protein; VO2 max: maximal oxygen uptake; IDF: International Diabetes Federation

Variables	Values, mean ± SD
Age (years)	49.5 ± 9.8
Weight (lbs.)	187.7 ± 44.9
Height (in)	68.3 ± 4.0
BMI (kg/m^2^)	28.14 ± 5.8
Systolic blood pressure (mmHg)	110.0 ± 12.7
Diastolic blood pressure (mmHg)	69.1 ± 8.6
Fasting glucose (mg/dL)	93.6 ± 16.4
Triglyceride (mg/dL)	129.5 ± 83.3
Total cholesterol (mg/dL)	192.5 ± 39.9
HDL cholesterol (mg/dL)	53.4 ± 15.6
LDL cholesterol (mg/dL)	113.2 ± 36.5
Metabolic syndrome (Criteria as defined by IDF guidelines)	0.9 ± 1.1
CRP (mg/L)	2.0 ± 2.0
Estimated VO_2 _max (ml.kg^-1^.min^-1^)	45.0 ± 9.2

**Figure 1:**
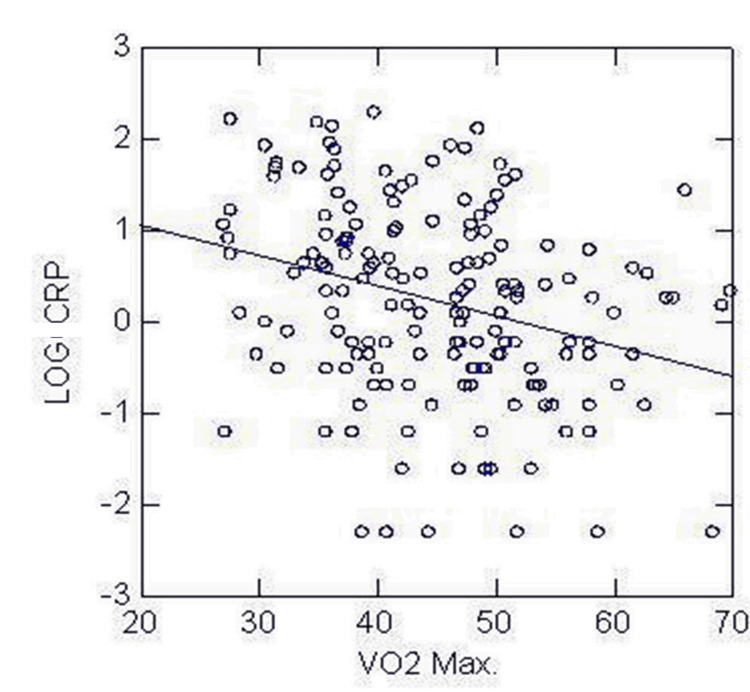
Inverse relationship between log CRP and VO2 max CRP: C-reactive protein; VO2: maximal oxygen uptake

In our study sample, 8.7% of the participants met the diagnostic criteria for MetS. Figure 1 shows the relationship between hs-CRP and estimated VO2 max. An inverse association was found between hs-CRP and estimated VO2 max (p < 0.01).

**Figure 2:**
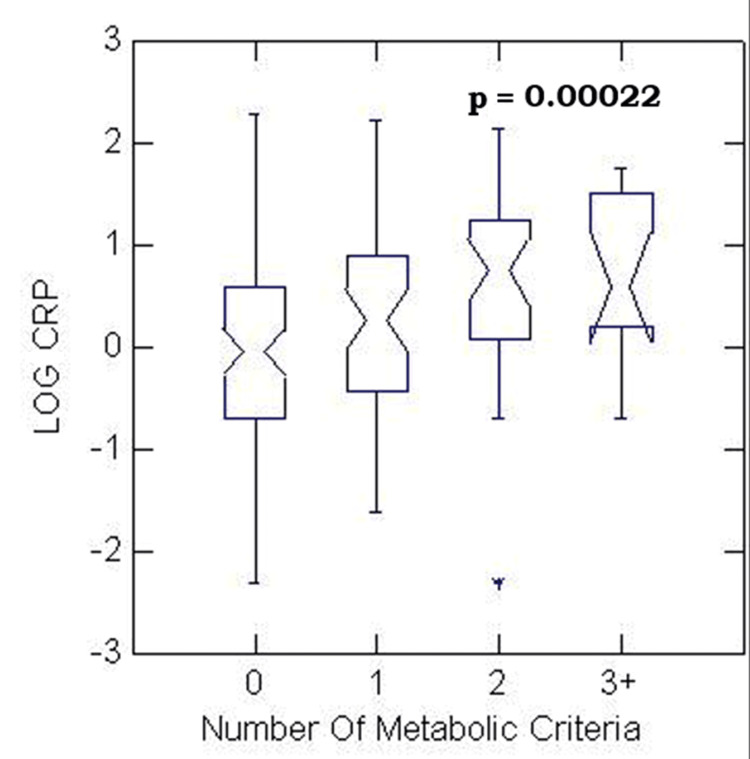
Distribution of log CRP levels among 173 participants according to the presence of 0, 1, 2, or ≥ 3 MetS criteria Box plots demonstrate median, 25th, and 75th percentile values of log CRP CRP: C-reactive protein; MetS: metabolic syndrome

As shown in Figure 2, hs-CRP increased linearly with the number of MetS criteria (p < 0.01). An inverse association was also found between the estimated VO2 max and the number of MetS criteria (p < 0.01) (Not shown). Estimated VO2 max was positively related to physical activity status (p < 0.01) (Not shown). Hence, fitness level was matched by the amount of reported physical activity.

## Discussion

The US Department of Health and Human Services (USDHHS) advises adults to engage in at least 150 minutes of moderate-intensity aerobic activity or 75 minutes of vigorous aerobic activity weekly to improve health and reduce the risk of chronic disease [16]. The American College of Sports Medicine (ACSM) recommends a minimum of 150 minutes per week of moderate-intensity exercise for health benefits [17]. Our findings support the USDHHS' physical activity recommendations and recommendations set by the ACSM. Subjects in our study engaging in two to three hours of exercise per week had hs-CRP levels ≤ 2.5 mg/L (p = 0.01817), considered low to moderate risk.

**Figure 3:**
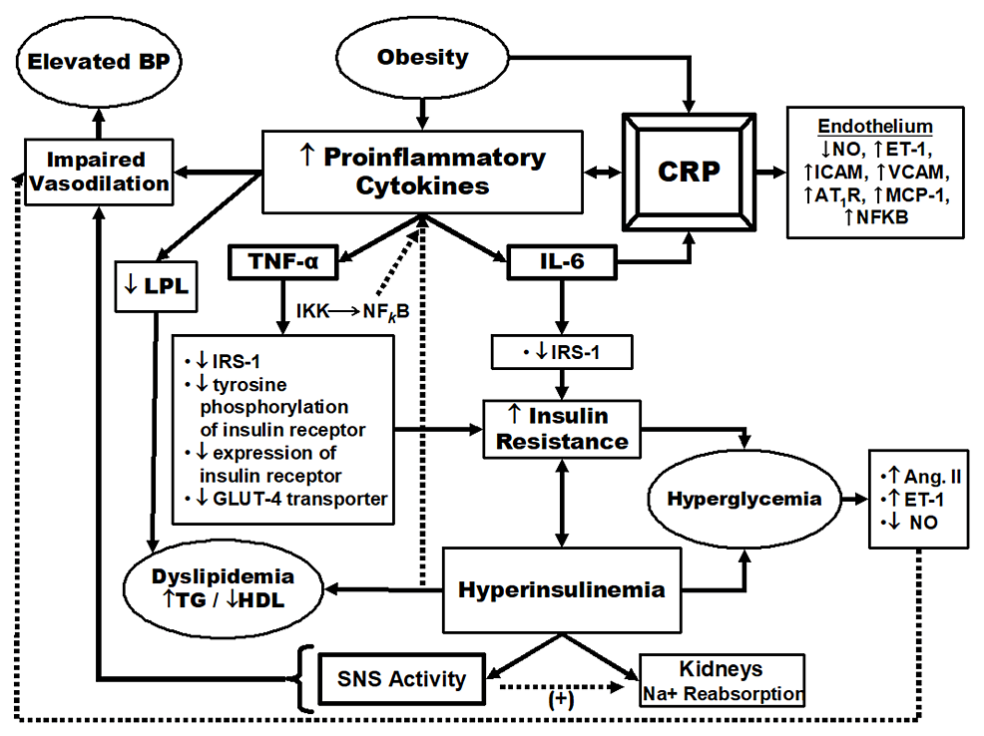
A model describing the relationship between inflammation and metabolic syndrome characteristics IL-6: interleukin-6; TNF-α: tumor necrosis factor-alpha; CRP: C-reactive protein; BP: blood pressure; TG: triglyceride IGT: impaired glucose tolerance; DM: diabetes mellitus; NO: nitric oxide; ET-1: endothilin-1; ICAM: intracellular adhesion molecule; VCAM: vascular cell adhesion molecule; AT1R: angiotensin type-1 receptor; MCP-1: monocyte chemoattractant protein-1; NFKB: nuclear factor KB; Ang. II: angiotensin type II; IRS-1: insulin receptor substrate 1; LPL: lipoprotein lipase; SNS: sympathetic nervous system Figure Credit: Authors

An apparent common theme connecting most MetS risk factors is inflammation (Figure 3) [18,19]. Inflammation plays a role in elevated BP, dyslipidemia, impaired glucose tolerance, and obesity [18,19]. The present study found a positive relationship between hs-CRP and MetS characteristics, similar to other studies [20,21]. We found an inverse association between hs-CRP levels and CRF, as measured by estimated VO2 max (p < 0.01). Additionally, we observed an inverse relationship between CRF levels and MetS severity, with estimated VO2 max decreasing as the number of MetS criteria increased (p < 0.01). It may be postulated that CRF reduces each risk factor seen in MetS via a reduction in the inflammatory process.

Several studies have found an inverse relationship between inflammatory markers and physical activity [8,10]. The pro-inflammatory cytokines, interleukin-6 (IL-6), and tumor necrosis factor-alpha (TNF-α) are involved in hs-CRP production. Regular exercise training has been observed to decrease basal plasma interleukin levels, including IL-6 and TNF-α [22]. This reduction subsequently leads to the downregulation of hs-CRP production. Prescott et al. found that increased transcriptional activity of TNF-α and IL-6 results in heightened intranuclear binding of the pro-inflammatory transcription factor known as nuclear factor *K*B (NF*K*B) [23]. Thus, it is suggested that exercise downmodulates activation of NF*K*B [24], thereby reducing TNF-α, IL-6, and CRP. This reduction in inflammation may, in turn, reduce risk factors seen in MetS. It is well-known that exercise improves BP [25]. This improvement is likely due to several mechanisms, including reduced cytokines and amyloid precursor proteins (APP). Hypertension (HTN) appears to stimulate the production of cytokines such as IL-6 and other inflammatory mediators from the endothelium. Studies indicate that heightened cytokine levels can initiate an acute phase reaction (APR), damaging endothelial cells. Elevated cytokines, along with APPs, may impair endothelial-dependent vasodilation [26]. Consequently, this damage may lead to the buildup of fatty plaques, narrowing the arteries and compromising blood flow. Moreover, chronic inflammation has been linked to impaired blood vessel dilation and the development of insulin resistance [27]. Insulin resistance syndrome can stimulate the brain to secrete more catecholamines, such as norepinephrine (NE), which can further enhance the APR and the inflammatory response [27]. This, in turn, can escalate the risk of HTN and atherosclerosis due to the endothelial damage resulting from these events. Another important MetS characteristic, impaired glucose tolerance, also improves with regular exercise. This improvement is likely due to several mechanisms, including reduced inflammation. Inflammation may activate NF-Kβ signaling pathways via up-regulation of TNF-α and IL-6, which stimulate hepatic CRP production [28]. TNF-α may then contribute to insulin resistance by increasing oxidation of free fatty acid (FFA), stimulating additional cytokines (i.e., IL-6), inhibiting the expression of GLUT-4 transporters, harming endothelial function, and/or impairing glucose-stimulated release of insulin by β-cells [28]. Hyperglycemia induces IL-6 from macrophages and endothelium [29]. Obesity is another risk factor seen in MetS. The beneficial impact of physical activity on this particular risk factor, as assessed through measurements such as BMI or body fat percentage, is widely recognized in scientific literature [27]. It may be that exercise reduces inflammation via a reduction in adiposity. IL-6 also induces the expression of cytokine signaling-3 (SOCS-3) suppressors, a negative regulator of insulin signaling [30]. Rehman et al. found that IL-6 levels are elevated in obese subjects with increased inflammation and insulin resistance [30]. Moreover, IL-6 may affect the function of pancreatic β-cells, which secrete insulin in response to glucose stimulation. This pro-inflammatory state may contribute to insulin resistance in obese individuals as well as those with MetS. 

The ensuing hyperinsulinemia stimulates IL-6 and TNF-α. Endothelial nitric oxide synthase-mediated vasodilation becomes impaired via the inflammatory cytokines and associated abnormal FFA oxidation. Reducing inflammation may be an important mechanism by which exercise improves glycemic control. Regular exercise has been found to increase the expression of GLUT 4 transporters [31] and the expression of insulin receptor substrate-1 (IRS-1) [32]. These alterations may be partly due to reductions in TNF-α and IL-6. Exercise has been found to improve glucose tolerance and both peripheral and hepatic insulin sensitivity [32]. These improvements will likely lead to reduced cytokine and APP release, improving glycemic control. 

The last MetS characteristic is dyslipidemia, which is also improved by exercise. Exercise, particularly aerobic exercise, appears to increase HDL and reduce TG [33,34]. In addition, sympathetically induced cytokines depress lipoprotein lipase (LPL) [27]. Thus, exercise appears to enhance the activity of several enzymes involved in lipid metabolism, including LPL [34]. Hence, exercise may enhance LPL activity via cytokine reduction, thereby reducing visceral adipose tissue and improving dyslipidemia.

As previously mentioned, exercise increases the expression of IRS-1 [35,36]. It may be that exercise enhances the expression of IRS-1 via a reduction in TNF-α and IL-6. Enhanced expression of IRS-1 may reduce interference in insulin's action and improve insulin sensitivity. It may be that exercise reduces pro-inflammatory cytokines and the inflammatory response via a reduction in body fat, particularly a reduction in visceral adiposity. This may be an important mechanism by which exercise reduces inflammation. It is recognized that adipose tissue functions in part as an immune organ. It secretes many immunomodulatory factors and sends inflammatory signals known to cause insulin resistance [37]. In adipose tissue, TNF-α reduces the expression of the insulin receptor and causes a reduction in tyrosine phosphorylation of the insulin receptor, thereby interfering with insulin action [38]. TNF-α also impairs insulin signaling through serine phosphorylation, leading to the development of T2DM. Anti-TNF-α treatment strategies have been developed to reduce the incidence of insulin resistance and T2DM. The intracellular pathways activated by TNF-α involve NF*K*B, which activates inflammatory target genes and inactivates the insulin receptor and IRS-1. This leads to decreased activation of phosphoinositol-3 kinase, a second messenger involved in the metabolic effects of insulin [38]. 

The potential impact of CRP on insulin sensitivity and production could be attributed to its ability to modulate the innate immune response, leading to heightened systemic inflammation [39]. IL-6 also interferes with insulin signaling by reducing the phosphorylation of insulin receptors and IRS-1 [30,38], which are essential for activating downstream pathways that mediate glucose uptake and metabolism. In animal models, TNF-α and IL-6 have been found to reduce LPL activity in adipose tissue [40]. 

The findings of this study underscore the importance of physical activity in managing MetS and provide valuable insights for healthcare professionals and policymakers alike. Healthcare professionals are crucial in promoting regular physical activity as a key component of MetS management. They should routinely assess patients' activity levels using validated tools and provide individualized counseling on increasing exercise, with specific and achievable recommendations based on the ACSM guidelines of 150+ minutes per week of moderate-intensity activity. Providers should address psychosocial and environmental barriers to exercise and help patients leverage their social support networks.

Translating these findings into practice will require a multi-faceted approach. Policymakers should support initiatives to increase population physical activity levels, especially among those at risk for MetS. At the individual level, this includes funding community programs offering low-cost exercise classes, healthy living workshops, and personalized coaching. Community-level efforts should focus on creating activity-promoting built environments through infrastructure investments, zoning regulations favoring mixed-use developments, and access to affordable exercise facilities. Within healthcare systems, integrating activity counseling into primary care visits for at-risk patients is key, enabled by provider training, quality measures, and reimbursement models incentivizing lifestyle interventions. A collaborative, multi-level approach is needed to create environments supporting regular physical activity to prevent and manage MetS and its health risks.

Limitations

This study has a few limitations. First, the cross-sectional design prevents establishing causality between CRF, inflammation, and MetS. Second, the study sample was recruited from a single center, which may limit the diversity of participants and the generalizability of the findings to broader populations with different demographic characteristics or health profiles. Third, the study included a relatively small sample size, which may limit the statistical power and reliability of the findings. While significant associations were observed between CRF, hs-CRP, and MetS, a larger sample size could provide more robust estimates and increase the likelihood of detecting smaller effect sizes. Future research should aim to recruit larger, more diverse samples to confirm and extend these findings.

Fourth, some data such as medical history and lifestyle practices were obtained through self-reported questionnaires. This introduces the possibility of recall bias or inaccuracies in data reporting, which could influence the study outcomes. Objective measures or medical records could be used in future studies to validate self-reported data. Fifth, while efforts were made to exclude participants with known cardiovascular diseases, t2DM, or other chronic conditions, there may still be unaccounted confounding variables that could impact the results. Factors such as diet quality, stress levels, sleep patterns, or undiagnosed health conditions could influence the relationships between CRF, inflammation, and MetS. Future research should aim to control for a wider range of potential confounders to strengthen the validity of the findings.

Additionally, BMI was used instead of waist circumference to assess obesity, and direct measurements of cytokines like IL-6 and TNF-α were not performed, limiting mechanistic insights. Medication use and comorbidities data were also unavailable, which could further confound the results. Future studies should incorporate more comprehensive body composition assessments, inflammatory markers, and relevant medical information to address these limitations.

## Conclusions

Elevated inflammation and MetS significantly increase the risk of CVD, DM, and premature mortality. This study's findings substantiate the beneficial effects of higher CRF and regular physical activity on reducing inflammation and ameliorating the risk factors associated with MetS. Our results demonstrate a clear inverse association between CRF, assessed by VO2 max, and hs-CRP levels, a widely recognized marker of systemic inflammation. Importantly, we observed that individuals engaging in two to three hours of exercise per week had hs-CRP levels within the low-to-moderate risk range (≤ 2.5 mg/L). This finding aligns with the physical activity recommendations set forth by authoritative bodies, such as the USDHHS and the ACSM*.*

This study is supported by compelling evidence for the beneficial effects of aggressive therapeutic interventions for these individuals. Our findings further underscore the importance of promoting regular exercise as a preventive strategy against the development of MetS, CVD, and T2DM. Future research should continue to explore the intricate mechanisms underlying these relationships and inform the development of targeted interventions for improving public health outcomes.
